# Wet lab and live surgical training at Aravind Eye Hospitals

**Published:** 2023-12-01

**Authors:** R Sankarananthan, Prasad R Senthil, Dhivya Ramasamy, Thulasiraj D Ravilla, Madhu Shekhar

**Affiliations:** 1 Medical Officer, Cataract and IOL Services: Aravind Eye Hospital, Madurai, India.; 2 Senior Faculty: Lions Aravind Institute of Community Ophthalmology (LAICO), Madurai, India.; 3 Executive Director: Lions Aravind Institute of Community Ophthalmology (LAICO), Madurai, India.; 4 Chief, Cataract and IOL Services: Aravind Eye Hospital, Madurai, India.

**Wet lab and live surgical training are both vital components of the residency programme in ophthalmology at Aravind Eye Hospitals in India.**

**Figure:**
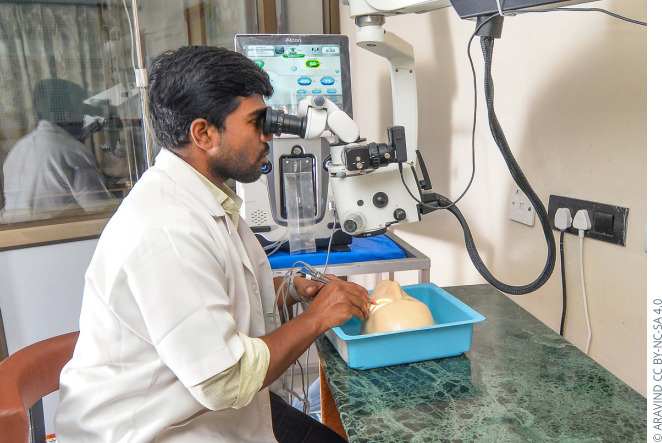
By using phaco machine in the wet lab, surgeons learn the principles of phaco while practising use of the foot pedal. **INDIA**

Before wet lab training begins, trainees attend lectures on the surgical anatomy of the eye, as well as intraoperative complications and their management. They watch detailed videos of each surgical step to get an in-depth understanding of the competencies required, the overall process, and the attitudes needed for managing outcomes.

The wet lab allows surgical trainees to become familiar with the handling of surgical instruments, develop hand–eye coordination, and practise surgical steps. Good wet lab training can improve the outcomes of operations performed by trainees.^1^

## Setting up the wet lab

An ideal wet lab simulates an ophthalmic operating room, where trainee surgeons can experience what it feels like operate on an eye. To keep costs down, refurbished equipment and instruments can be used.

A basic ophthalmic surgical wet lab includes the following:

- Operating microscope (fitted with a camera and screen for external viewing)
- Ophthalmic surgical instruments (refurbished, or re-sterilised ‘single use’ instruments)
- Animal or human cadaver eyes, and devices to mount them
- Phaco machine (refurbished).

Training is overseen by a designated trainer, and surgeons practice while being assisted by an experienced operating theatre nurse.

### Practising on goat eyes

A goat eye is preferred in the wet lab as its anatomy and tissue texture closely resembles that of the human eye. When using goat eyes, ensure that the globe is not perforated and the cornea does not become dry.

**Figure 1:**
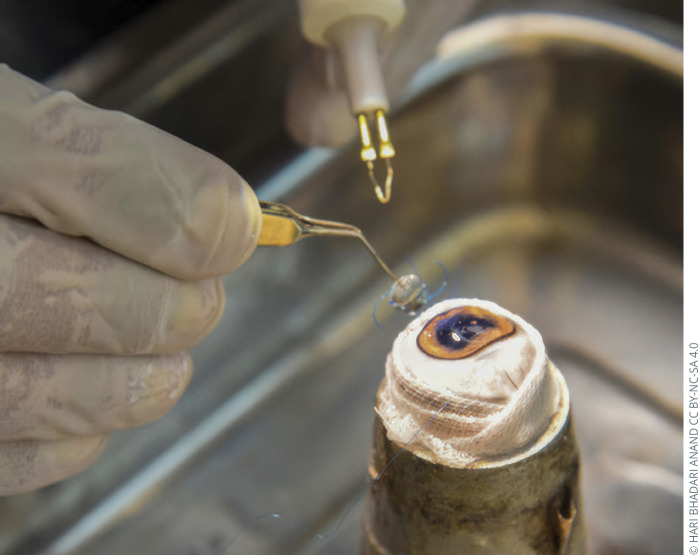
A goat's eye held in place using gauze.

The goat eyeball is mounted onto a metal stand using a roll of gauze, wrapped around the equator so the eyeball snuggly fits on the stand (Figure 1). Next, we inject intravitreal formalin to form a firm globe.

Before wet lab training begins, trainees attend lectures on the surgical anatomy of the eye, as well as intraoperative complications and their management. They watch detailed videos of each surgical step to get an in-depth understanding of the competencies required, the overall process, and the attitudes needed for managing outcomes.

The wet lab allows surgical trainees to become familiar with the handling of surgical instruments, develop hand–eye coordination, and practise surgical steps. Good wet lab training can improve the outcomes of operations performed by trainees.^1^

Trainees can practise creating sclerocorneal tunnels on four sides of the globe, and can practise capsulorrhexis and hydrodissection. However, a goat eyeball offers limited scope for practising nucleus prolapse and delivery.

To prepare a goat eye for phacoemulsification practise, cover the cornea in wet gauze to prevent drying and then place the eye in a combination microwave oven, set to 100°C, for four seconds. This renders the nucleus hard and leathery, and allows trainees to practise trenching, dividing, chopping, and emulsification of the nucleus.

In settings without access to a microwave oven, we create a sclerocorneal tunnel, implant a nucleus that was extracted during manual small-incision cataract surgery, and suture the tunnel closed. Phacoemulsification can then be practised on this nucleus.

Goat eyeballs can also be used to practise trabeculectomy and implantation of glaucoma drainage devices, in addition to cataract surgery.

It may be possible to use human cadaveric eyes (that are unfit for transplantation), to practise keratoplasty. It is part of the scrub nurse's role to prepares the cadaveric eyes and ensure proper disposal of the eyeballs afterwards.

## Other ways to practise

The polyurethane foam sheets in which ophthalmic sutures are packaged can be used to practise suturing and making sclerocorneal tunnels.

The thin polythene film that is sometimes used in the packaging of intraocular lenses can of the used to practise capsulorrhexis. A circle of 6 to 7 mm is marked over the polythene sheet with a pen, and trainees can use a cystotome to practise rhexis within the mark. This allows for multiple attempts, which cannot be done on a cadaver eye.

Once a trainee has made 20 sclerocorneal tunnels on goat cadaveric eyes, completed 100 attempts at capsulorrrhexis on polythene film, and 20 sets of sutures in polyurethane foam sheets, they are permitted to move on to live surgical training.

## Live surgical training

Each group of trainee surgeons is assigned to an experienced surgeon, who will be responsible for training them. During the first four to five days, trainees are expected to closely observe while the senior surgeons operate on patients. They are also expected to learn how to handle different instruments, maintain aseptic precautions, and establish the correct physical posture during surgery.

Following the observation period, live hands-on training begins on selected patients: those with uncomplicated immature cataracts and who have a well-dilated pupil, normal anterior chamber depth, and no ocular comorbidity. Surgical training rooms are equipped with observation facilities for trainees and the trainer: either microscopes with an observer-scope and/or a camera and a monitor for viewing.

The training is administered in a step wise fashion: trainees can progress to the next step after performing the previous step satisfactorily. For instance, a trainee will be allowed to perform capsulorrhexis only after performing sclerocorneal tunnels satisfactorily.

Patient safety is a priority. So, if a trainee faces difficulty in any step, the surgery is immediately taken over by the trainer.

## Feedback and review

The trainer and trainees review the recorded surgery videos and detailed feedback is given on the same day as the surgery. Objective scoring of the surgery is done using the ICO-Ophthalmology Surgical Competency Assessment Rubric (ICO-OSCAR) in the trainee's log book.^2^ Intraoperative complications and any difficulties faced during a surgical manoeuvre are also noted in the log book.

## Monitoring post-operative outcomes

The effectiveness of any surgical training programme is judged by the post-operative outcomes of trainee surgeons. It is mandatory for all trainees to do post-operative assessment of the operations earlier in the day, together with their trainer. The log books are also evaluated by a senior consultant, who provides further insights into the surgical skills that a trainee should acquire.

**Figure 2:**
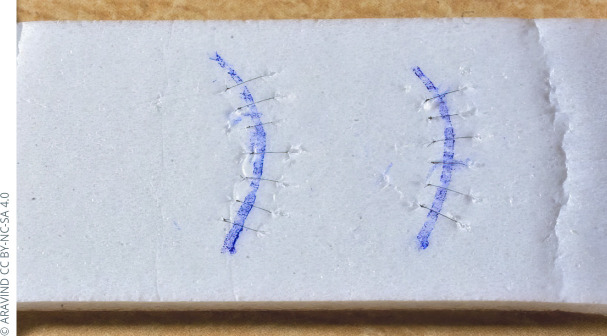
Polyurethane foam sheets can be used to practise suturing.

**Figure 3:**
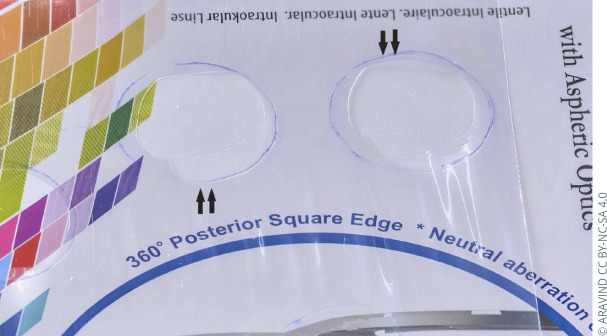
Capsulorrhexis can be practised on a polythene sheet.

Aravind Eye Hospitals’ CATQA (Cataract Quality Assurance) system tracks long term surgical outcomes.^3^ Quarterly complication review meetings are held where surgeons with high complication rates are identified and given focused re-training going back to wet lab and live training to fine tune specific aspects of their surgery. Although it applies to any surgeon, most re-training happens in newly certified surgeons. It has been estimated that surgeons need to perform manual small-incision cataract surgery an average of 300 times before their complications levels stabilise at acceptable levels.^4^

Trainees who perform exceptionally well are provided with higher order surgical skill training, such as tackling complex cataracts and managing intraoperative complications.

It is vital for a training centre to establish structured surgical training and set up tools to assess the effectiveness of training strategies. The learning curve may not be uniform among trainees; hence it is imperative to periodically monitor the surgical performance data of amateur surgeons so that focused re-training can be accomplished. Surgeons who perform well can be promoted to undertake high-volume surgery and manage complex surgical scenarios.

**Table 1:** Tips for learners and educators.

Tips for learners	Tips for trainers
Have a thorough understanding of surgical anatomy before starting surgical training.	Assess the surgical knowledge of a trainee before initiating training so that you know what level of training is needed.
Assess each surgical step as it is being performed. This will help you to identify and prevent complications.	Do not take for granted any aspect of the procedure. Ensure that you teach every detail of each surgical step.
Pay close attention to each surgical step because it has a direct bearing on the outcome of the next step.	Teach good surgical habits right from the start of the training period.
Complications are an inherent part of surgical training. Be open about your concerns and discuss them with senior colleagues who can help you to improve your skills.	Immediately correct any minor deviation from the preferred technique.
